# Survival outcomes of CD34^+^CD38^−^LSCs and their expression of CD123 in adult AML patients

**DOI:** 10.18632/oncotarget.26118

**Published:** 2018-09-25

**Authors:** Asmaa M. Zahran, Sanaa Shaker Aly, Amal Rayan, Omnia El-Badawy, Maged Abdel Fattah, Arwa Mohammed Ali, Hala M. ElBadre, Helal F. Hetta

**Affiliations:** ^1^ Clinical Pathology Department, South Egypt Cancer Institute, Assiut University, Assiut, Egypt; ^2^ Clinical and Chemical Pathology Department, Faculty of Medicine, South Valley University, Qena, Egypt; ^3^ Clinical Oncology Department, Faculty of Medicine, Assiut University, Assiut, Egypt; ^4^ Medical Microbiology and Immunology Department, Faculty of Medicine, Assiut University, Assiut, Egypt; ^5^ Medical Oncology Department, South Egypt Cancer Institute, Assiut University, Assiut, Egypt; ^6^ Medical Biochemistry Department, Faculty of Medicine, Assiut University, Assiut, Egypt

**Keywords:** acute myeloid leukemia, CD34+CD38-LSCs, CD34+CD38-CD123+LSCs, disease free survival, overall survival

## Abstract

**Background and aim:**

Acute myeloid leukemia (AML) is one of the most common leukemias in adults. AML is generally regarded as a stem cell disease characterized by an accumulation of undifferentiated and functionally heterogeneous populations of cells, The aim of the present study was to identify leukemia stem cells in patients with AML and their correlations with treatment outcomes namely remission status, disease free survival, and overall survival.

**Results:**

The mean percentages of CD34^+^CD38^-^ and CD34^+^CD38^low/−^CD123^+^ LSCs were 2.2± 0.4and 22.3± 2.6, respectively. The percentages of CD34^+^cells, CD34^+^CD38^-^ and CD34^+^CD38^low/−^CD123^+^ LSCs were significantly lower in AML patients with complete remission than those without complete response (*P*<0.001, *P*<0.004, *P*<0.001 respectively). The mean OS of all study patients was 20.03±1.2 months while the median OS was 21 months (95% CI=18.32-21.48). The mean DFS was 16.96±1.02 months and the median was 18 months (95% CI=8.9-11.4). DFS and OS were significantly higher among those who achieved CR than those without CR. In addition, there were significant negative effects of WBCs, CD34^+^cells, CD34^+^CD38^-^ and CD34^+^CD38^-^CD123^+^LSCs on DFS and OS.

**Patients and methods:**

We investigated 30 patients with newly diagnosed AML; all patients underwent complete history taking, and thorough physical and clinical examination, complete blood count. Peripheral smears and bone marrow aspirates were also examined. Cytochemistry and immunophenotyping of leukemic cells were performed routinely in bone marrow using monoclonal antibodies. Flow cytometry was used to analyze leukemia stem cells and their expression of CD123.

**Conclusion:**

Our study elucidated that CD34+CD38-LSCs, with or without CD123+LSCs phenotype was present in a significant proportion of AML patients and it could be responsible for resistance to traditional treatments, and high percentage of MRD that was translated into significantly high number of non CR, poor DFS, and OS.

## INTRODUCTION

Acute myeloid leukemia (AML) is one of the most common leukemias in adults. AML is generally regarded as a stem cell disease characterized by an accumulation of undifferentiated and functionally heterogeneous populations of cells [1, 2]. AML stem cells are CD34 positive cells; in addition they have been recognized as CD38 negative [3]. These CD34+CD38-cells have chemotherapy-resistant properties [4] and are therefore possibly responsible for the outgrowth of minimal residual disease (MRD), which may cause relapse. Treatment failure in AML may be caused by the presence of LSCs [5]. LSCs exist in a stem cell microenvironment, also known as a stem cell niche, in a quiescent state, which allows the LSCs to become chemo resistant [6–7].

To recognize and identify LSCs and their behavior that may play an important role in the approach of targeted therapy. LSCs are considered as CD34+CD38− population and often express CD123, CD44, or CD184, which are rarely expressed on normal hematopoietic stem cells and could be also potential therapeutic targets [8–12]. CD123, the interleukin-3 (IL-3) receptor-alpha subunit, is highly expressed on LSCs [13]. Permanent cure of acute AML by chemotherapy alone remains elusive for most patients because of the inability to eradicate effectively LSCs. By identification of LSC specific cell surface markers, we can distinguish them from normal hematopoietic stem cells, so that, helps effectively to develop therapies targeting them [14]. A better understanding of LSCs and their molecular biology will allow the design of more effective therapies [15]. Although advances in the treatment of AML have led to survival improvement for younger patients, but this is not true for elderly patients where about 70% of patients 65 years or older die of AML within one year.

The aim of the present study was to identify LSCs in patients with AML and their correlations with treatment outcomes namely remission status, disease free survival (DFS), and overall survival (OS).

## RESULTS

The present study was carried out on 30 patients with denovo AML, 16 males and 14 females. Their ages ranged from 21 to 67 years with median age 46 years. Demographic, clinical and laboratory data of patients with AML were demonstrated in (Table 1, 2).

**Table 1:** Demographic data of AML patients

Item	Patients (30)
Age	46±2.10
Sex	16/14
(male/female)	53.33%/ 46.67%
WBCs (10^9^/L)	31.15±4.46
Platelets (10^9^/L)	36.57±3.54
Hemoglobin (gm/dl)	7.46±0.35
Bone marrow blast (%)	62.08±4.14
Peripheral blood blast (%)	31.42±1.24

Data represented as mean ±SE, mean percentage (blasts) ± SE, WBCs: white blood cells.

**Table 2:** Clinical and laboratory characteristics of 30 AML patients

Characteristic	Descriptive
CD34^+^LSCs	49.48±4.06
CD34^+^ CD38^low/−^CD123+ LSCs	22.25±2.59
CD34^+^ CD38^low/−^LSCs	2.23±0.38
FAB classification	
M0	0 (0%)
M1	1 (3.3%)
M2	14 (46.7%)
M3	8 (26.7%)
M4	4 (13.3%)
M5	3 (10%)
Response	
Remission	21 (70%)
Not in remission	9 (30%)

Abbreviations:AML: Acute myeloid leukemia.

Data expressed as mean percentage ±SE, numbers, and percentages.

The mean percentage of blast cells in BM of AML patients was 62.08± 4.14 with a range of (24 - 95%) and in peripheral blood was 31.42±1.24 with a range of (11.3-53.3). The mean percentage of CD34 expression in the blast cells was 49.48± 4.06 with range (2 - 92%), while the mean percentage of CD34^+^CD38^-^ leukemic stem cells was 2.23± 0.38 with a range of (0.7-9.3%), and the mean percentage of CD34^+^CD38^low/−^CD123^+^ cells was 22.25± 2.59 with a range of (1-46.2%) as shown in (Table 2).

Twenty one of AML patients (70%) achieved complete remission (CR) after induction chemotherapy. while nine patients (30%) did not achieve complete remission (non –CR). When the demographic and hematological parameters among CR and non- CR groups were compared (Table 3). There was no statistical significant difference regarding age, hemoglobin and platelets count between CR and non CR groups. While white blood cell count and BM blasts were significantly decreased in CR group than non-CR group (*P*<0.001 and *P*<0.005 respectively). In addition to that, the percentage of CD34+ leukemic cells, CD34^+^CD38^-^ leukemic cells and CD34^+^CD38^low/−^CD123^+^ cells were also significantly reduced in AML patients with CR than those with non-CR (*P*<0.001, *P*<0.004, *P*<0.001 respectively).

**Table 3:** Relations between response and other variables

Variable	Not in remission (9)	Remission (21)	P value (<0.05)
FAB			P=0.357n.s
M1	0.0	1(4.8%)	
M2	5(55.0%)	9(42.9%)	
M3	2(22.2%)	6(28.6%)	
M4	0.0	4(19.0%)	
M5	2(22.2%)	1(4.8%)	
Blast	78.93±2.26	54.85±20.01	P<0.005^**^
WBCs	51.72±21.19	22.33±20.37	P<0.001^***^
CD34^+^	69.22±28.16	41.01±12.23	P<0.001^***^
CD34^+^ CD38^low/−^LSCs	4.85±1.44	2.53±1.95	P<0.004^**^
CD34^+^ CD38^low/−^CD123^+^LSCs	34.73±7.18	16.90±13.13	P<0.001^***^
HB	7.50±2.25	7.45±1.87	P=0.943n.s
PLTs	38.90±22.14	35.57±18.60	P=0.674n.s

Abbreviations: n.s: non-significant, P-value significant<0.05; PLTs: platelets; HB: hemoglobin, WBCs: white blood cells.

Chi^2^ test, Unpaired *t*-test and Mann Whitney test were used for significance, ^**^ indicates moderately significant, ^***^indicates highly significant.

### Survival analysis among the study group

**Figure 1:**
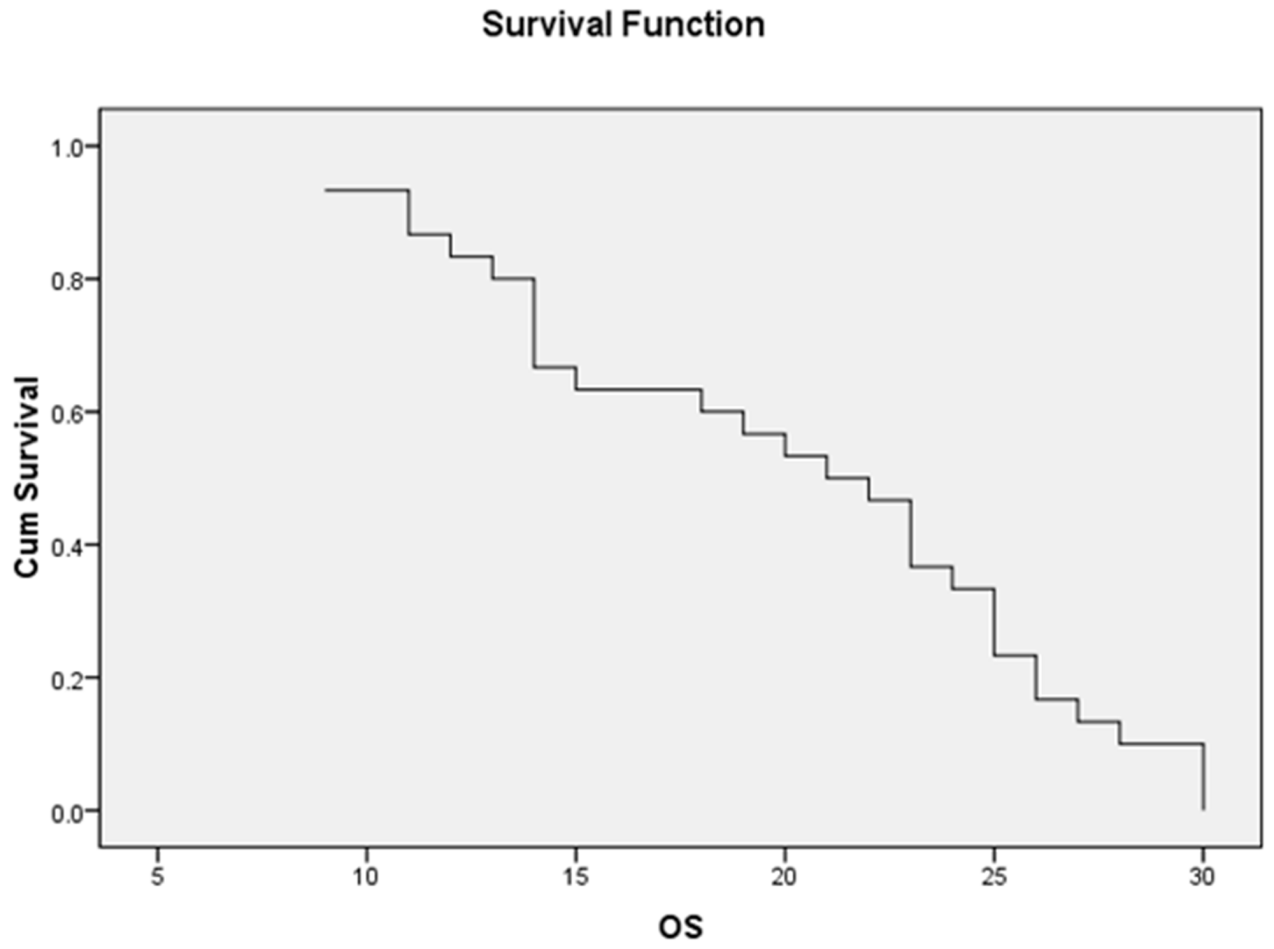
The median OS of the whole AML patients was 21 ms (95% CI=18.32-21.48)

**Figure 2:**
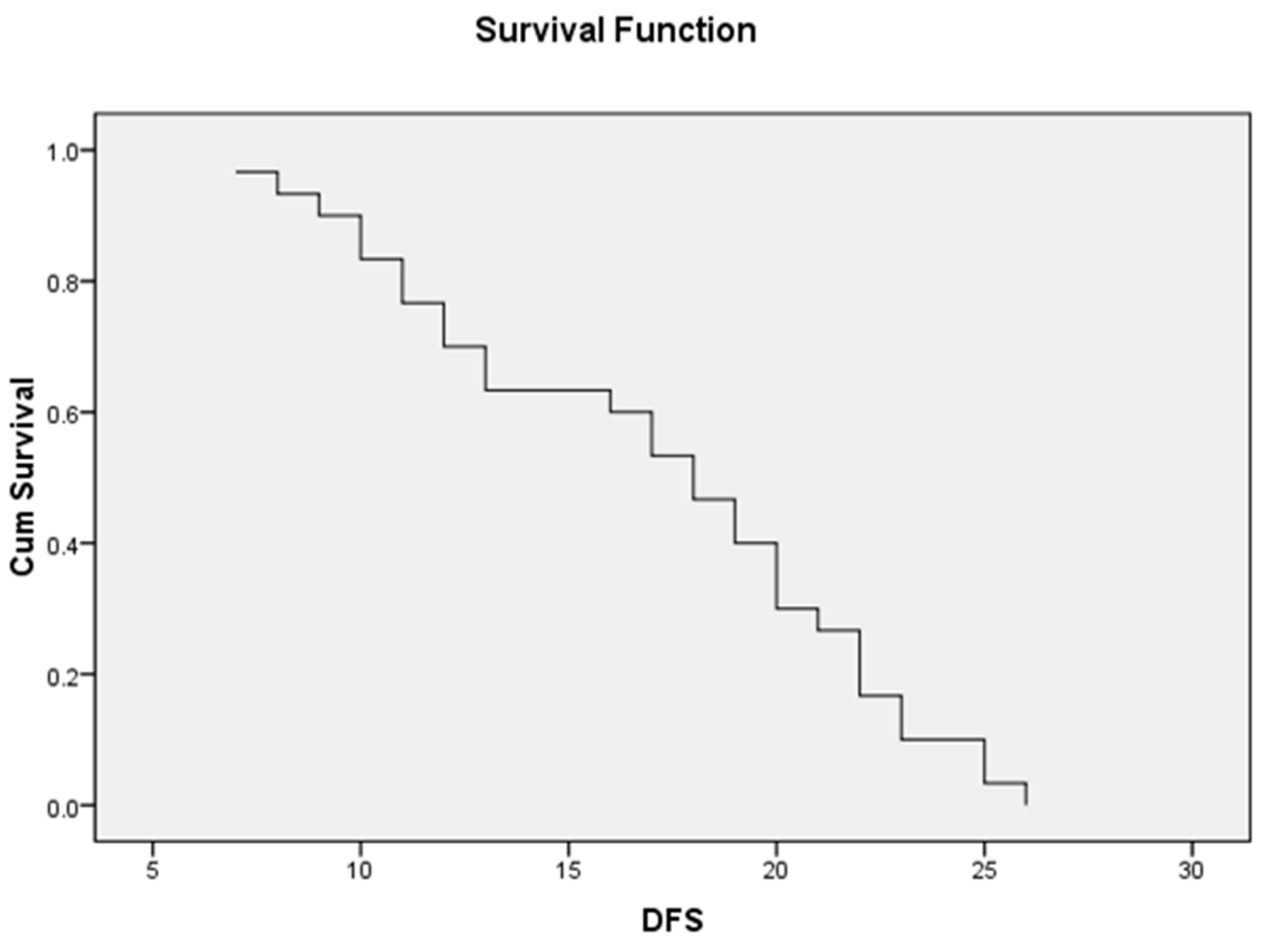
The median DFS of 30 AML patients with a median of 18 ms (95% CI=8.87-11.35)

The mean OS of the whole study patients was 20.03±1.22 months while the median OS was 21 months (95% CI=18.32-21.48) (Figure 1), moreover, the mean DFS was 16.96±1.02 months and the median was 18 months (95% CI=8.8-11.6) (Figure 2).

**Figure 3:**
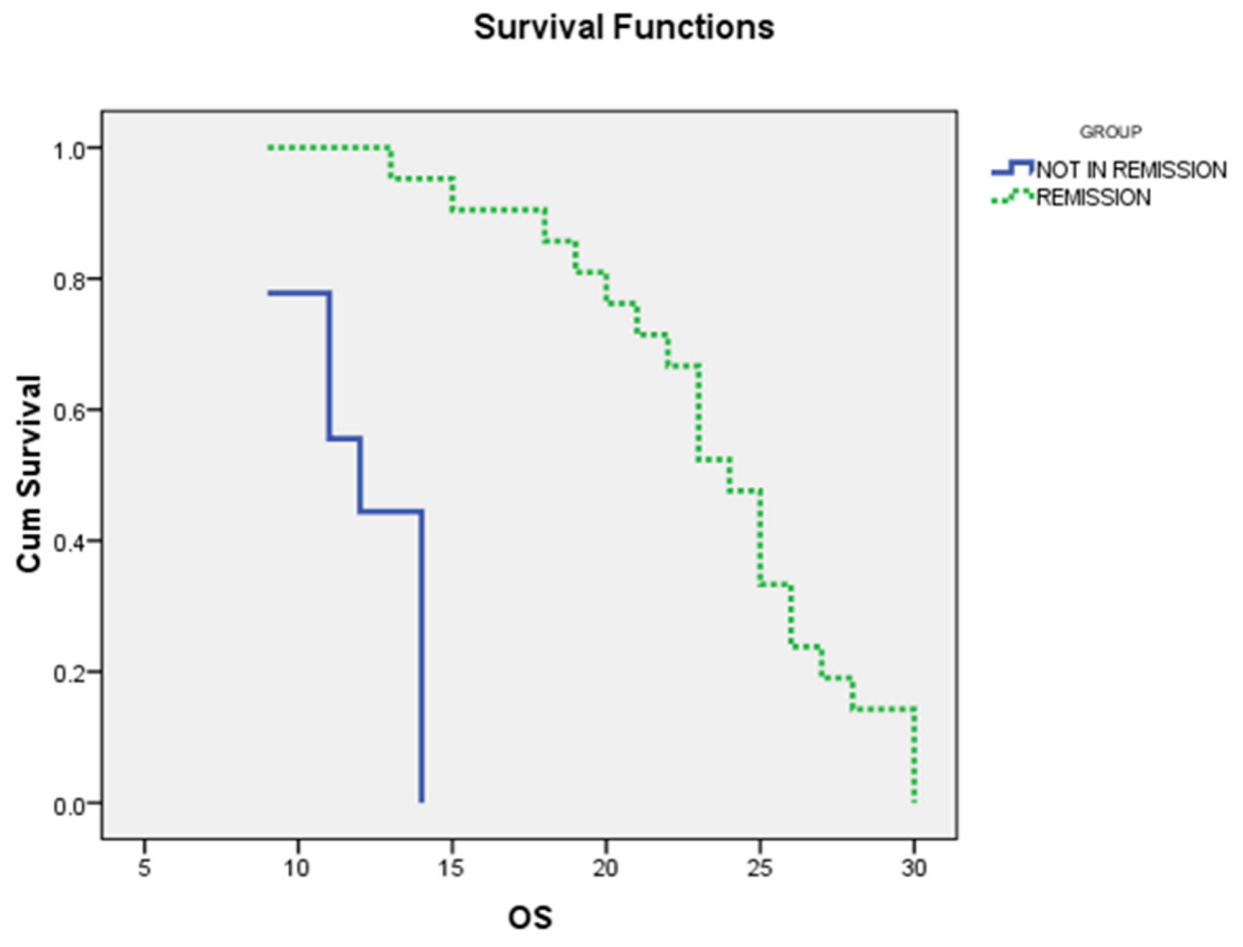
The Overall survival (OS) curves of AML patients with and without complete remission (CR): the median OS of AML patients with CR was 24 months, while for those with non CR was 12ms (P<0.000)

**Figure 4:**
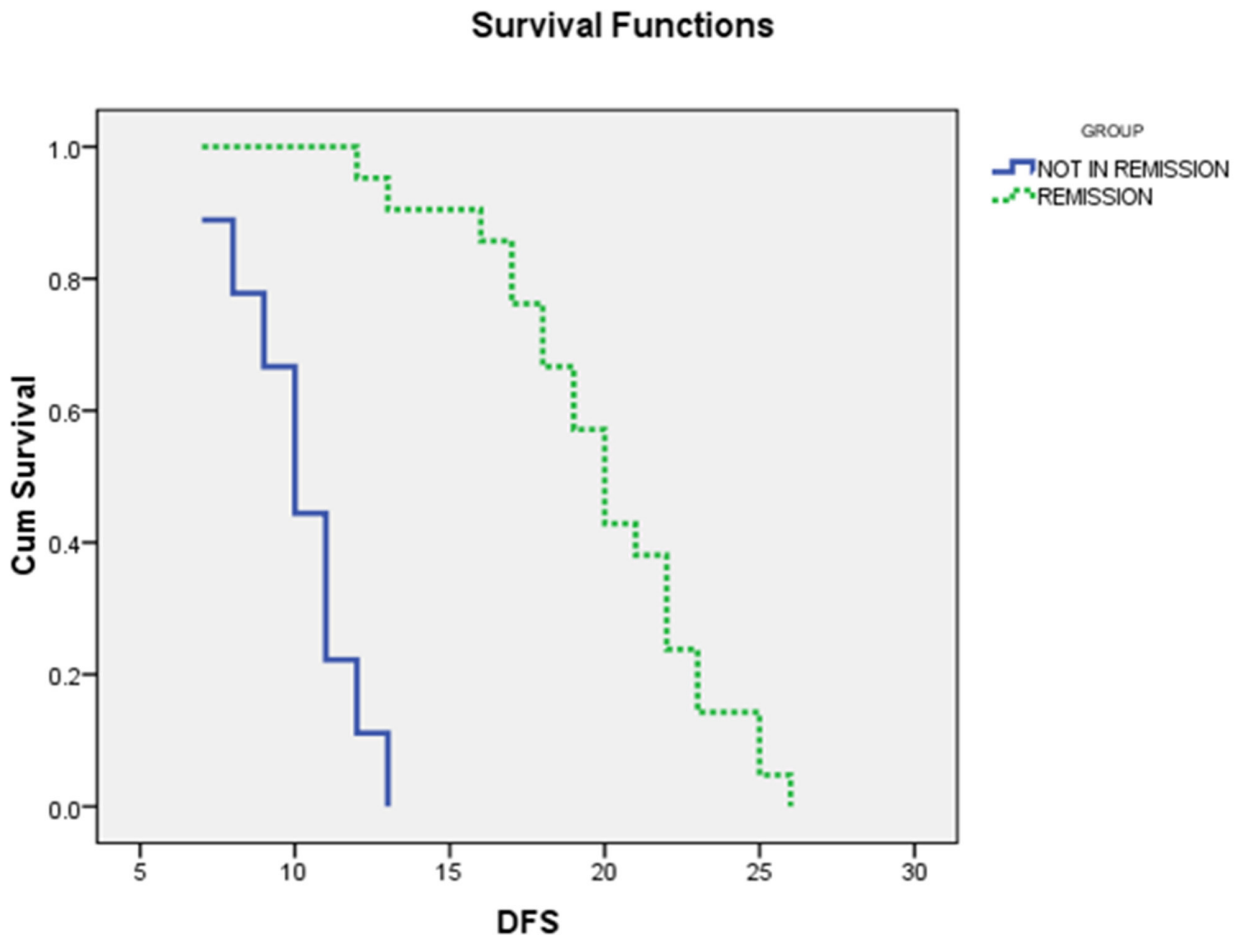
The Disease free survival curves of AML patients with and without complete remission (CR): the median DFS for AML patients with CR was 20 months, while for those with non CR was 10 months, P<0.000

As shown in (Table 4) and (Figures 3, 4), the mean OS of AML patients with complete remission was significantly higher than those who failed to achieve complete remission (23.5±1.01 vs. 10±0.7, *P*<0.000) and the median was (24 vs. 12 months, respectively). Similarly, the mean DFS of AML patients with complete remission was significantly higher than those who failed to achieve complete remission (10.1±0.6 vs.19.9±0.9, P<0.000) and the median was (20 vs. 10 months, respectively).

**Table 4:** Relations between treatment response and survival time

Item	Not in remission “n=9”	Remission “n=21”	p-value <0.05
**1- DFS**			
“mean ±SE”	10.11±0.633	19.90±0.81	P<0.000^***^
Median	10.0 ms	20.0 ms	
**2- OS**			
“mean ±SE”	10.00±0.70	23.48±1.01	P<0.000^***^
Median	12.0 ms	24.0 ms	

Abbreviations: DFS: disease free survival; OS: overall survival; n: number; SE: standard error, P-value<0.05.

^***^indicates highly significant.

There were significant negative correlations between WBCs, CD34^+^LSCs, CD34^+^ CD38^low/−^LSCs, CD34^+^ CD38^low/−^CD123^+^LSCs and DFS, also significant negative correlations between the same factors and OS implicating difficulty of salvage therapy or reinduction therapy to prolong survival in this group of AML patients, correlations couldn’t be done between FAB type and survival (because of small number of patients in each type and different types of variables as FAB is categorical variable while survival is numeric one), as shown in (Table 5).

**Table 5:** Correlations between survival time and different prognostic factors among 30 AML patients

Prognostic factor	DFS (N=30)		OS (N=30)	
	r	P value	R	P value
HB level	0.169	0.371 n.s	0.200	0.289 n.s
WBCs count	-0.728	0.000 h.s	-0.586	0.001^***^
Platelets count	0.030	0.873 n.s	-0.067	0.725 n.s
Blasts count	-0.281	0.133 n.s	-0.297	0.111 n.s
CD34^+^LSCs	-0.562	0.001 m.s	-0.541	0.002^**^
CD34^+^ CD38^low/−^LSCs	-0.363	0.049 s	-0.398	0.029^*^
CD34^+^ CD38^low/−^CD123^+^LSCs	-0.489	0.006 m.s	-0.489	0.005^**^

Pearson correlation was used, and *T*-test for significance with P value considered significant at <0.05, n.s; nonsignificant, ^*^indicates significant, ^**^indicates moderately significant, ^***^indicates highly significant.

## DISCUSSION

LSCs are known by their ability to undergo self-renewal and their capacity to differentiate [16, 17]. For clinical treatment and patient survival it is important to know which putative LSCs will survive therapy. In that respect it is important to realize that the CD34^+^CD38^-^ compartment has been shown to be the most therapy resistant *in vitro* [18].

In this study, we found that the mean proportion of CD34^+^ CD38^low/−^LSCs was statistically significantly lower in patient with acute leukemia in CR compared to patients with non-CR and this agreed with Hwang and colleague, who had demonstrated in a group of 54 AML patients, that the proportion of CD34^+^ CD38^low/−^cells at diagnosis was significantly lower in patients achieving CR compared to patients who did not achieve CR [19]. Also our results were in agreement with the results of Wilson et al. and *l*e Viseur et al. [20–21] who reported that there was a correlation between CD38 expression and MRD status, so that AML patients with leukemic blasts expressing CD38 were more likely to be MRD positive.

In our study, we found that the percentage of CD34^+^ CD38^low/−^CD123+ cells (CD123^+^ LSCs) was significantly lower in patient with AML with CR than in patients with non-CR and this result came in harmony with the study of Hwang et al., [19] who observed a somewhat higher level of CD123 expression on LSCs in the non-CR group and relapsed group. Also this agreed with *Jin et al.*, who observed that the increased levels of CD123^+^ LSCs in AML patients were known to be associated with a high proportion of blasts and a low CR rate [22].

The burden of LSCs in AML patients is considered a strong biomarker for clinical outcomes in AML [23–24]. CD34^+^ CD38^−^LSCs frequency is associated with worse outcomes namely remission and survival [5]; AML patients with greater than 3.5% of CD34^+^CD38^-^ LSCs show a median relapse free survival of 5.6 mo vs. 16 mo in those with a lower percentage of CD34^+^CD38^-^ cells [5]. Our results were in agreement with the previous studies as the high frequency of CD34^+^ CD38^low/−^LSCs was translated into no remission and poor DFS and OS.

Several studies confirmed that CD34^+^ CD38^-^ cell population had a prognostic impact on survival. The median event-free survival (EFS) was 8.2 months (3-year EFS: 29%) for those with a higher percentage of CD34^+^ CD38^-^ versus 91.9 months (3-year EFS: 62%) for those with a lower percentage [25], our results were comparable with these studies where DFS was significantly impaired by the large number of CD34^+^ CD38^low/−^LSCs in our patients.

Our results proved that higher number of WBCs was significantly associated with shorter EFS and OS and this came in agreement with other studies [26]. This significant negative correlation between higher WBC counts and reduced OS and DFS could be explained partly by the fact that high WBC counts were associated with an increased risk of tumor lysis syndrome and leukostasis and both were considered oncologic emergencies and subsequently affected the prognosis of patients [27].

The presence of CD123 on AML CD34^+^/CD38^−^ cells has a potential significance. It is demonstrated that LSCs are biologically distinct from their normal stem cell counterparts. As CD123 is not found on normal HSCs, it may provide a unique marker for identification this malignant clone. This feature may be very useful in minimal residual disease studies as a single and standardized marker [28].

The percentage of the CD34^+^ CD38^low/−^ population at diagnosis strongly correlates with clinical outcome, in addition, survival and outgrowth of leukemia cells after therapy may depend on many factors including LSC load [24]. This supported our results that detected a negative impact of the percentage of CD34^+^ CD38^low/−^ LSC subpopulation on remission status and survival.

CD34^+^CD38^-^CD123^+^ cells represent highly resistant population to chemotherapy and might be responsible for regrowth of leukemia and thus relapse of the disease as many studies proved that high level of CD34^+^CD38^-^CD123^+^ LSCs are predictive of poor EFS and OS [5, 29], collectively our results were in concordance with these studies.

Generally, eradication of blasts can be achieved by systemic multi-agent chemotherapy. However given that LSCs contained within CD34^+^CD38^-^CD123^+^ population in AML and difficulty to eradicate these malignant cells in this subgroup supports the hypothesis that in order to maintain complete remission after induction chemotherapy and to improve survival in AML patients, traditional chemotherapy targeting blasts should be combined with agents targeting LSCs.

With the poor prognosis of AML and only little improvements in treatment options, there is an imperative need for novel therapies, so therapies targeting LSC may offer a hope for prognostic improvement. In this regard, selection of the target highly expressed by LSCs and the timing of therapy may result in improved prognosis.

## PATIENTS AND METHODS

In our study, we investigated 30 patients with newly diagnosed AML attending to South Egypt Cancer Institute, from May 2016 to October 2017. The study protocol followed the ethical guidelines of the 1975 Helsinki Declaration and was approved by the Ethical and Research committee of South Egypt Cancer Institute, Assiut University. An informed written consent in accordance was taken from all cases. Diagnosis of AML was achieved by morphologic, cytochemical, immunophenotypic and cytogenetic studies.

**All patients were subjected to:**

• Complete history taking, thorough physical and clinical examination.
• Complete blood count (CBC) including differential leukocyte count was done Using cell dyne 3500 automated cell counter (ABBOTT DIAGNOSTIC).
• Peripheral smear examination and bone marrow aspiration examination.
• Cytochemistry studies, such as myeloperoxidase, esterases, acid phosphatase and Periodic acid–Schiff.
• Immunophenotyping of leukemic cells was performed routinely in bone marrow using monoclonal antibodies that were used for diagnosing AML included: CD34. CD13, CD33, CD117, CD15 and intracellular myeloperoxidase, CD14, HLA-DR, CD41, CD61 and anti-glycophorin A. All monoclonal antibodies were purchased from Becton Dickinson (BD) Bioscience, CA, USA.
• Flow cytometric detection of LSCs and their expression of CD123
• Induction chemotherapy after diagnosis given for those younger than 60 years was in the form of anthracycline days 1-3 (Daunorubicin 60-90 mg/m^2^ or Idarubicin 12 mg/m^2^) and Cytarabine 100-200 mg/m^2^ continuous IV days 1-7 ± Cladribine 5 mg/m^2^ days 1-5. While for patients 60 years or older; the previous regimen was given for those with good performance; unfavorable cytogenetics or molecular markers; antecedent hematologic disorder; or therapy-related AML. And lower intensity therapy was given for those not candidate for intensive therapy like Cytarabine 20 mg/m^2^ SC twice daily days 1-10, and others.

### Flow cytometric detection of CD34^+^ CD38^-^ LSCs and CD123^+^ LSCs

**Figure 5:**
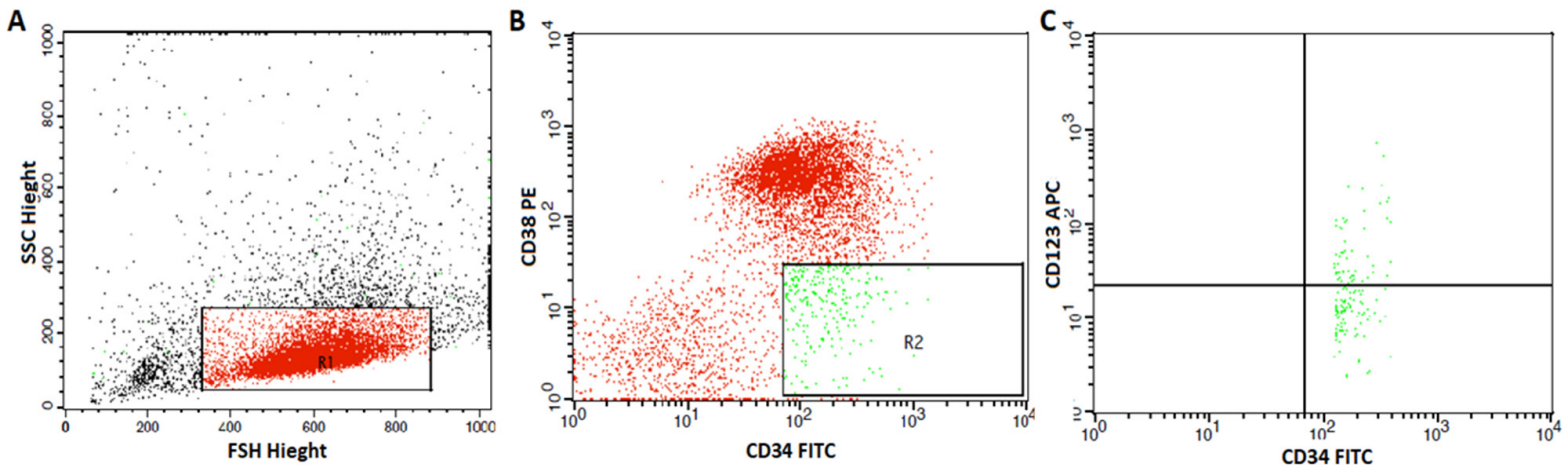
Flow cytometric detection of leukemic stem cells and their expression of CD123 **(A)** Forward and side scatter histogram was used to define the blast cells. **(B)** The expression of CD38 and CD34 were assessed on the blast cells and gate were drown to identify CD34+CD38-cells. **(C)** Then, the expression of CD123 were assessed on CD34+CD38-cells.

Ethylenediaminetetra-acetic acid (EDTA)-anticoagulanted fresh bone marrow (BM) aspirates under complete aseptic conditions from patients with AML were collected at the time of diagnosis to measure the LSCs using FACS caliber flow cytometer (Becton Dickinson, San Jose, CA, USA). Fifty μL from BM was stained with 5 μL of phycoerythrin (PE) conjugated anti CD38, fluoroisothiocyanate (FITC) conjugated anti CD34 (Bioscience, USA) and allophycocyanin (APC) conjugated anti-CD123 (all from BD Bioscience, CA, USA) and incubated in dark for 15 min in dark at room temperature. After incubation, red blood cells (RBCs) lyses and washing with phosphate buffered saline were done. Acquisition and analysis were performed using cell Quest soft ware. Leukemic blasts were gated in on the basis of characteristic forward and side scatter features. Then the expressions of CD34 and CD38 markers were evaluated in the leukemic blasts to detect CD34+CD38- leukemic cells. Then the expression of CD123 marker was evaluated on CD34+CD38- LSCs. CD34+CD38- LSCs and CD34^+^CD38^−^CD123^+^ cells (CD123^+^ LSCs) were expressed as a percentage of total blast cells (Figure 5).

### Statistics

Data were presented as mean ±SE, percentages. For the association between quantitative variables and categorical dependant variables (remission, no remission) unpaired *t*-test test and Mann Whitney test were used, furthermore, Chi^2^ test was used to assess the relations between 2 categorical variables (response and FAB subtype), Pearson correlation was used to study the magnitude of association between different prognostic factors and survival.

Regarding the analysis of OS, and DFS of our patients, for the whole study group survival curves were constructed by Kaplan-Meier method, for comparison of DFS, and OS between two unpaired groups (remission, no remission) log rank test was used and differences with P-value <0.05 being considered significant with 95% confidence interval (95% CI), P-value <0.01-0.001 was considered moderately significant, and P-value<0.001 was considered highly significant.

The definitions used for calculation of survival followed the revised recommendations of the international working group for therapeutic studies [30]. OS was defined as the time interval between date of diagnosis and date of death or last follow up visit, while DFS was defined as the time interval between date of diagnosis and date of relapse, induction failure, or death from any other cause.

Remission was defined as the absence of signs and symptoms of the disease associated with normal CBC, and less than 5% blasts in BMA. Deviation from these criteria of remission was considered no remission. All data were analyzed using SPSS version 21.

## CONCLUSIONS

Our study elucidated that the CD34^+^ CD38^low/−^CD123^+^ phenotype is present in a significant proportion of AML patients and it may be responsible for resistance to traditional treatments, and high percentage of MRD that is translated into significantly high number of non CR, poor DFS, and OS.
